# Recent Prospective in CAR T-Based Therapy for Solid and Hematological Malignancies

**DOI:** 10.3390/biomedicines11020627

**Published:** 2023-02-20

**Authors:** Hany E. Marei, Carlo Cenciarelli

**Affiliations:** 1 Department of Cytology and Histology, Faculty of Veterinary Medicine, Mansoura University, Mansoura 35116, Egypt; 2 Institute of Translational Pharmacology (IFT)-CNR, 00133 Rome, Italy

Given that CAR-T cell therapy is effective in CD19-positive blood malignancies, it offers great hope for a variety of aggressive tumors that have thus far shown very little response to available therapies. The basis of CAR-T cell treatment is the use of patient-specific T cells that have been genetically modified to express an antibody fragment with a single chain variable (scFv) that is specific to a tumor-associated antigen and is connected to intracellular co-stimulatory molecules. Target cells are killed when CAR-T cells engage with tumor cells, causing cytotoxicity and the release of several inflammatory cytokines. Despite the enormous potential of CAR-T cell-based therapy, several obstacles remain, including the heterogeneity of cancer cells, the aberrant signaling pathways implicated in tumor progression, antigen escape, the hostile suppressive tumor microenvironment, and dysfunctional or exhausted T cells. Before an entire clinical application can commence, these problems must be addressed.

A different T cell therapy has gained popularity lately, and we can expect that this Special Issue will present advancements in T cells that have been modified with Fcγ-chimeric receptors (CRs) that are directed against solid tumors and blood malignancies. The engagement of Fcγ-CRs on the surface of effectors cells with the Fc region of tumor-bound antibodies can promote tumor elimination by antibody-dependent cellular cytotoxicity (ADCC).

We also plan to highlight the most recently completed and ongoing clinical trials with an emphasis on the justification for using various CAR-T cell types and highlighting the various ways in which such trials are designed to overcome particular challenges, as well as how outcomes reflect the improvement of therapeutic arsenals against various cancer types.

This Research Topic includes 10 manuscripts that highlight current knowledge and future directions for the potential therapeutic of CAR-T cells for solid and hematological malignancies. 

A switch receptor (SwR) was created when the extracellular domains of the TGFβ receptor and the intracellular domains of the IL-2/15 receptor are fused. The authors assessed SwR in conjunction with two anti-STEAP1 CAR versions, a protein that is highly expressed in prostate cancer. A panel of STEAP1+/ prostate cancer cell lines with or without the over-expression of TGFβ or additional TGFβ was used to test the SwR-CAR T cell’s activity. In terms of T cell proliferation and survival, cytokine response, and cytotoxicity, the SwR-CAR constructions enhanced the functionality of CAR-T cells in TGFβ-rich settings. The SwR-CAR-T cells maintained their STEAP1-specific activity while developing an activated effector memory state [1]. 

Protein transduction domain (PTD)-IVT-mRNAs were created using a novel delivery platform to express CAR in NK-92 cells. Two different ErbB(+) cancer cell lines were used to test the efficacy of CAR T1E-engineered NK-92 cells, which carry the sequence of T1E single-chain fragment variants (scFv) to detect the ErbB receptor and either contain CD28 or 4-1BB as co-stimulatory signaling domains. NK-92 cells were successfully transduced with the CAR’s PTD-IVT-mRNA and allowed to express it. In the co-incubation experiments, CAR T1E-engineered NK-92 cells caused high levels of cell death (25–33%) against both HSC-3 (oral squamous carcinoma) and MCF-7 (known as breast adenocarcinoma) human cells [2]. 

AdCAR, a versatile adaptor CAR-T cell technology that enables multitargeting. Three separate B-lineage lymphoma cell lines were screened, and the results showed unique immune target characteristics. In CD19-negative lymphoma subsets, CD19 CAR-T cells typically lost their ability to function; however, AdCAR-T cells could be redirected to additional target antigens besides CD19, such as CD20, CD22, CD79B, and ROR-1. The ability to change the adaptor molecule’s specificity allows for an extended range of applications and considerably boosts anti-leukemic and anti-lymphoma activity [3]. 

The efficacy and safety of intraperitoneal (IP) versus intravenous (IV) CAR-T cell therapy in the treatment of high-grade serous ovarian cancer (HGSOC) were studied. A CAR that targets the ErbB2/HER2 protein (ErbB2CAR), which is overexpressed in HGSOC, has been developed, and its therapeutic potential evaluated on ovarian cancer cell lines (OVCAR8, SKOV3, and NAR). Compared to an IV injection, IP-injected ErbB2CAR-T cell therapy in tumor-bearing mice led to disease remission and improved survival. Furthermore, IP-injected ErbB2CART cells circulate less, making them less dangerous for organs other than tumors. These findings highlight the advantages of an IP CAR-T cell administration over an IV one, providing not only a safer approach but also the ability to mitigate the impact of ErbB2CAR in HGSOC [4]. 

An overview of different approaches to current anti-FLT3 CAR-T cells under development was provided. Although further studies are still required to assess their safety profile, these therapies are frequently effective both in vitro and in vivo [5]. 

CAR-T cells targeting roundabout guidance receptor 4 (Robo4), which is expressed at high levels in tumor vascular endothelial cells, by incorporating three anti-Robo4 single-chain variable fragments (scFv) that were identified using phage display were developed [6]. In in vivo studies on mice carrying B16BL6 melanoma, all three T-cells were shown to suppress tumor growth, and this activity was linked with Robo4 binding affinities. However, according to the authors, tumor regression was also seen when CAR-T cells were given to L1.2 cancer-bearing mice that expressed mRobo4 but which had the lowest Robo4 affinity. The authors conclude that there are still many unsolved problems; however, in the future, in vitro and in vivo functional binding assessments will be required to gather fundamental data to understand how CAR-T cells are effectively tuned.

An overview of the state-of-the-art developments in CD19 auto-CAR-T cell treatment, the management of particular toxicities, and the role of allogeneic CAR-T development in this context were provided. Modern frontline chemoimmunotherapy regimens can cure more than half of non-Hodgkin lymphomas (NHL); however, the prognosis for patients with relapsed and/or refractory (r/r) illness is still poor, especially if they are not candidates for hematopoietic stem cell transplantation. As a result, r/r NHLs are a population with significant medical needs. For patients with r/r B cell malignancies, CD19-directed autologous chimeric antigen receptor (auto-CAR) T cells have had a revolutionary impact. A total of four CAR-T cell products will receive regulatory approval from the U.S. Food and Drug Administration (FDA) between 2017 and 2021 due to the significant response rates and durable remissions that have been attained in this scenario [7]. 

A list of locoregional therapy of potential CAR-T cell delivery methods, systemic administration with and without targeted ultrasonography, direct intra-arterial drug delivery, and nanoparticle-enhanced delivery in glioma as prospective routes of administration were provided. The study discussed published, planned, and ongoing clinical trials using CAR-T cell therapy for malignant glioma. The authors reported that increasing neoadjuvant and/or adjuvant combinatorial immunotherapeutic concepts and modalities with specific modes of action for malignant glioma meant that selecting administration routes would become increasingly important [8].

As a result of recent developments in CAR technology, customized CARs such as multi-specific and logic-gated CARs have been created. NK cells and macrophages are two other immune cell types modified to express CARs to cure cancer. Additionally, novel techniques for treating autoimmune illnesses and other disorders and diseases have been developed using CAR technology. Recent developments in alternative CAR therapies and design, mechanisms of action, application difficulties, and potential future paths were reviewed [9].

A succinct overview of the history of CAR, including its uses in various tumors, MSCs’ special capacity as a delivery vector for cancer therapy, and the potential to boost the activity of CAR-immune cells, were all provided by Chan et al. (2022). They have emphasized how both therapies work well together. Numerous CAR-T/-NK cell clinical trials that are still in progress, as well as the commercial success of CAR-related products such as Kymriah (Novartis) and Yescarta, make clear the substantial breakthrough that has been made in CAR technology (Kite Pharma, Gilead). Mesenchymal stem cell engineering is a crucial form of cell therapy (MSC). MSCs have been investigated as potential therapeutic agents due to their intrinsic homing capabilities onto tumor locations and their release of cytokines that combine CAR and MSC technologies. As an immunotherapy intervention, this combination enables medicines to overcome their respective shortcomings [10]. 

## Data Availability

All data are available in the manuscript.
